# Supplementary material for: Dynalign II: common secondary structure prediction for RNA homologs with domain insertions
Source: Nucleic Acids Res. 2014 Nov 21;42(22):13939–48. doi: 10.1093/nar/gku1172 (PMC4267632; doi:10.1093/nar/gku1172)
Supplement: SUPPLEMENTARY DATA [file supp_gku1172_nar-02021-z-2014-File012.zip › manual/javadoc/RNAstructure_java_interface/source/package-summary.html]

RNAstructure\_java\_interface.source


JavaScript is disabled on your browser.


- Overview
- Package
- Class
- Tree
- Index
- Help

- Prev Package
- Next Package

- Frames
- No Frames

- All Classes

# Package RNAstructure\_java\_interface.source

A package that encapsulates all the Java classes that comprise the RNAstructure Java interface.

See: Description

## Package RNAstructure\_java\_interface.source Description

A package that encapsulates all the Java classes that comprise the RNAstructure Java interface.
  
  
There are no classes in this package.
Instead, all Java interface classes are in the subpackages of this one.
This is because the interface is backed by C++ classes, and these C++ classes are separated from the Java classes in the RNAstructure\_java\_interface folder.
C++ classes cannot be documented with Javadocs, but are documented with Doxygen formatting.

- Overview
- Package
- Class
- Tree
- Index
- Help

- Prev Package
- Next Package

- Frames
- No Frames

- All Classes
